# Lateral palatal flap approach to the nasopharynx and parapharyngeal space for transoral robotic surgery: a cadaveric study

**DOI:** 10.1007/s11701-012-0351-6

**Published:** 2012-04-24

**Authors:** Raymond K. Tsang, Catherine Mohr

**Affiliations:** 1 Department of Surgery, Division of Otolaryngology-Head and Neck Surgery, University of Hong Kong, Queen Mary Hospital, 102, Pokfulam Road, Hong Kong, SAR, China; 2 Medical Research, Intuitive Surgical Inc., Sunnyvale, CA USA; 3 Stanford University School of Medicine, Palo Alto, CA USA

**Keywords:** Robotic surgery, Nasopharyngectomy, Recurrent nasopharyngeal cancer

## Abstract

The da Vinci surgical robot has been used for minimally invasive surgery of the head and neck region including resection of tumors in the nasopharynx. Access to and vision of the nasopharynx with the robot are difficult. A pure transoral approach and midline palatal split approach have been described. The disadvantage of these approaches is the limited lateral access to the parapharyngeal space. The objective of this study was to investigate the feasibility of accessing the nasopharynx and parapharyngeal space with a lateral palatal flap. Two complete nasopharyngectomies with resection of the parapharyngeal space and exposure of the internal carotid artery and branches of the mandibular nerves were performed on two fresh cadavers with the da Vinci surgical robot. The set up of the robot, the surgical procedure of elevating the lateral palatal flap, and robotic resection of the nasopharynx and parapharyngeal space are described.

## Introduction

The surgical robot has led to many possibilities for minimally invasive surgery. In the head and neck region, the surgical robot is now used for resection of early tonsil cancer and for performing thyroidectomies without a neck scar. The surgical robot with its excellent 3D vision and the superior manipulation of the EndoWrist^®^, enables dissection of tissue in tight spaces, for example the pelvis and oral cavity. The nasopharynx, situated in the center of the head, is regarded as a difficult surgical site by traditional approaches. Various approaches have been described but most external approaches are complicated, and cross a substantial amount of normal tissue. The endoscopic approach has also been described but manipulation of endoscopic instruments is limited, and limits the approach to resection of small centrally located lesions. The surgical robot circumvents the difficulty of instrument manipulation in the tight space and should be applicable to resection of lesions in the nasopharynx and adjacent parapharyngeal space. Preclinical studies have demonstrated the feasibility of en bloc resection of the nasopharynx [1, 2]. There are also two case reports on the use of robotic surgery for resecting recurrent nasopharyngeal carcinoma [3, 4].

Currently there is no established approach for robotic resection of the nasopharynx. In this study, we investigated the feasibility of approaching the nasopharynx via a lateral palatal flap for robotic resection of the nasopharynx, the adjacent lateral parapharyngeal soft tissue, and the medial pterygoid muscle.

## Materials and methods

The study was performed in the laboratory of Intuitive Surgical, the manufacturer of the da Vinci Robotic Surgical System (Sunnyvale, CA, USA). Robotic nasopharyngectomy with resection of the adjacent parapharyngeal soft tissue and the medial pterygoid muscle was performed on two fresh cadavers.

### Raising the palatal flap

**Fig. 1:**
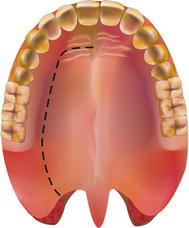
Schematic diagram of the incision for the lateral palatal flap

**Fig. 2:**
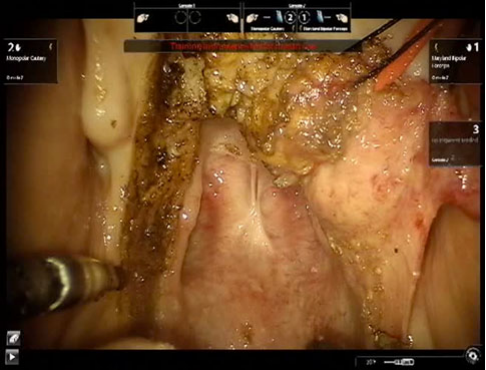
View of the nasopharynx after retraction of the palatal flap to the contralateral side

**Fig. 3:**
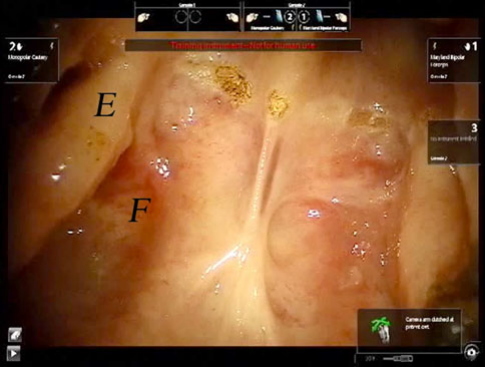
Close up view of the nasopharynx. *F* denotes the fossa of Rosenmuller. *E* denotes the posterior cushion of the Eustachian tube

The cadaver was positioned supine with the head flexed. The operating table was tilted ten degrees, head up, to increase the range of motion of the camera arm. The mouth was opened with a Boyle–Davis gag and secured with a Mayo table. The patient cart was place above the head of the cadaver with the endoscope arm in the midline. A 30° endoscope was used and a 5 mm monopolar cautery spatula was placed on the left robotic arm, and an 8 mm bipolar Maryland dissector was used in the right arm. An incision was made in the lateral hard palate mucosa on the right, just medial to the upper alveolus from the level of the incisor foramen extending posteriorly through the greater palatine foramen to the lateral soft palate, until it reached the anterior tonsillar pillar at the level of the upper pole of the tonsil. Figure 1 shows a schematic diagram of the incision. The palatoglossus muscle was identified and separated from the medial pterygoid muscle. The palatoglossus muscle was then divided sharply. The tensor veli palatini tendon was divided just medial to the hamulus of the medial pterygoid plate. The hard palate mucosa was elevated from the hard palate bone and the soft palate was detached from the posterior edge of the hard palate from the right edge and extended through the midline to the left side. The left greater palatine vessels were kept intact, because this is necessary blood supply for the mucosal flap. The soft palate was elevated with the Maryland dissector and carefully separated from the lateral wall of the right nasopharynx and anterior cushion of the right Eustachian tube with the cautery spatula. Two stitches were then placed on the edge of the flap to retract the flap to the left side. After completion of the palatal flap, the entire posterior nasopharyngeal wall, right Eustachian tube, and right fossa of Rosenmuller can be visualized. Anteriorly the posterior nasal cavity and the entire posterior choana could be well visualized and the robotic instruments could reach into the nasal cavity. Figure 2 shows the view of the posterior nasopharyngeal wall and posterior choana after retraction of the palatal flap to the left side. Figure 3 shows the close-up view of the nasopharynx including the right Eustachian tube and right fossa of Rosenmuller.

### Robotic nasopharyngectomy

**Fig. 4:**
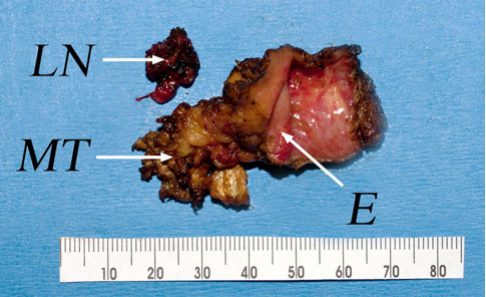
Photograph of the resected specimen. *E* denotes the right Eustachian tube opening. *MT* denotes the upper part of the right medial pterygoid muscle. *LN* is the resected parapharyngeal lymph node

Index cuts were made with cautery to mark the resection limits. The anteriosuperior limit of resection was the junction of the posterior choana with the roof of nasopharynx, and the left lateral resection limit was just medial to the left fossa of Rosenmuller. The inferior resection limit was down to the level of the upper pole of the tonsil. The resection started inferiorly, to divide the nasopharyngeal mucosa through the superior constrictor muscle and the longus capitus muscle down to the bone. This incision was carried out laterally to divide the medial pterygoid muscle. The attachments of the pharyngobasilar fascia and superior constrictor were divided from the medial pterygoid plate. The origin of the medial pterygoid muscle was then detached from the lateral pterygoid plate. The entire inferior nasopharynx with the medial pterygoid muscle and paraparhygeal fat was dissected from the underlying C1 and clivus. Finally, the cartilaginous Eustachian tube and the attachment of the levator palatine and tensor veli palatine muscles were divided at the skull base level and the whole specimen was resected en bloc. The resected specimen is shown in Fig. 4.

**Fig. 5:**
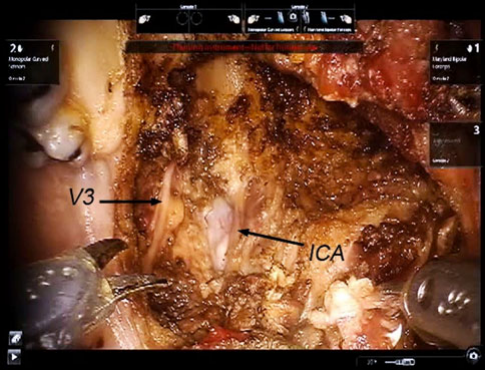
Exposure of the right internal carotid artery (*ICA*) and branches of right mandibular nerve (*V3*) after resection

After removal of the specimen, gentle blunt dissection around the carotid sheath just lateral to the longus capitus muscle exposed the internal carotid artery. The branches of the V3 can be seen lying on the lateral pterygoid muscle (Fig. 5).

### Insetting of a fasciocutaneous flap to the nasopharynx

**Fig. 6:**
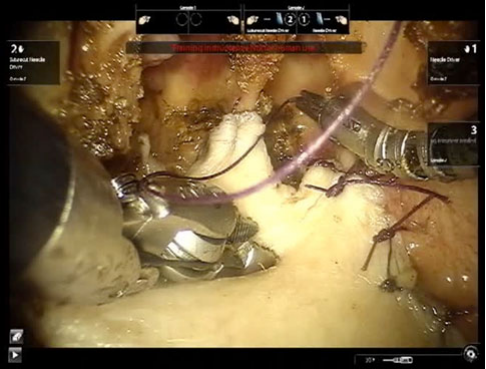
Suturing of the fasciocutaneous flap to the nasal cavity and nasopharynx by use of robotic instruments

To investigate the feasibility of insetting a fasciocutaneous flap to the nasopharynx to cover the raw bone and the exposed internal carotid artery, a piece of skin with subcutaneous fat and fascia was harvested from the chest wall. The size of the fasciocutaneous flap was 4 cm × 4 cm. The flap was placed on the raw area of the nasopharynx and sutured to the mucosa edges with 3–0 vicryl sutures (Johnson and Johnson, NJ, USA). A 5 mm needle driver and an 8 mm suture-cut needle driver (Intuitive Surgical) were used to perform interrupted stitches and knot tying. Figure 6 is a video capture of the robotic arms performing suturing of the flap to the posterior nasal cavity.

### Closure of the palatal flap

Holes were drilled in the posterior edge of the bony hard palate and vicryl sutures were passed through the holes to attach the soft palate to the hard palate with submucosal stitches. The cut in the palatoglossus muscle was repaired with 3–0 vicryl. The soft palate incision was closed with 3–0 vicryl interrupted sutures but the anterior hard palate incision was not sutured. For live patients, a dental plate would be used to splint the hard palate mucosa and enable the incision to heal, as described by Ng [5].

## Results

The setup time for the procedure was 20 min. Raising of the palatal flap required another 20 min. The palatal flap approach enabled excellent exposure of the whole nasopharynx, posterior choana and, more importantly, the ipsilateral parapharyngeal fat and soft tissue. The robotic arms enabled en bloc resection of the nasopharynx with the cartilaginous Eustachian tube cartilage, parapharyngeal fat, and medial pterygoid muscle. The carotid artery was not exposed during the procedure but the vessel may be readily dissected out without injuring the vessel wall. Finally, it was feasible to suture a piece of fascia or fasciocutaneous flap to the nasopharynx for coverage of the raw area. The robotic arms enabled placement of sutures in the tight spaces of the nasopharynx and posterior choana. The resection time was 30 min and the suturing time was 1 h. The time taken for closure of the palatal wound was another 45 min.

## Discussion

Operations on the nasopharynx are regarded as difficult, because of the inaccessibility of the region. Multiple external approaches have been designed in the past but all these approaches require crossing and disruption of a large amount of normal tissue and, in many approaches, multiple osteotomies [6–9]. The efficacy of the maxillary swing approach in salvaging recurrent nasopharyngeal carcinoma after radiation has been well documented for a large series by Wei et al. and also for smaller series in other institutes [10–12]. The disadvantage of this approach is that it requires facial incision and multiple osteotomies, which have their own morbidities and healing problems. With the advent of endoscopic sinus surgery, other surgeons sought to adapt these techniques to nasopharyngeal surgery and reported their experience with endoscopic approaches [13, 14]. The main reported limitation of the endoscopic approach was the limited manipulation of current endoscopic instruments in the narrow space of the nasopharynx. Manipulations such as suturing and knot tying were very difficult with the endoscopic approach and alternative techniques, for example endoscopic staplers for endoscopic sinus surgery, were not available.

The da Vinci surgical robot has led to new opportunities for minimally invasive surgical approaches to the nasopharynx, which should overcome the limitations of open and previously described endoscopic approaches. The three-dimensional magnified endoscopic view provided by the robot enables excellent vision of the operative field, and the EndoWrist^®^ of the surgical robot enables an unparalleled range of motion in a tight space. Previous reported approaches for robotic nasopharyngectomy were either transnasal or central palatal split or a combination of both. We decided to try a lateral palatal flap, because nasopharyngeal carcinomas are usually not centrally located and prone to lateral extension to the parapharyngeal space. The new AJCC staging in 2010 actually reclassified the T2 stage as parapharyngeal extension [15]. The central palatal split approach, while adequate for resection of centrally located tumors, may encounter limitations when resecting lesions lateral in the fossa of Rosenmuller and parapharyngeal fat space. The soft palate attached to the lateral nasopharyngeal wall may limit the lateral view and manipulation of the robotic arms. To circumvent these limitations of robotic arm movement, Dallan et al. [2] described their experience with the suprahyoid cervical port. Our objective with the lateral palatal flap approach was to improve the lateral view of, and instrumentation in, the parapharyngeal space. Our experiment showed that the lateral palatal flap approach results in an excellent view of the lateral nasopharyngeal wall and we were able to remove all the parapharyngeal fat and the medial pterygoid muscle to expose the mandibular branch of the trigeminal nerve lying on the lateral pterygoid muscle without requiring a suprahyoid cervical port.

Lateral extension of a nasopharyngeal carcinoma frequently abuts the internal carotid artery in the post-styloid space. Resection of a tumor that abuts the internal carotid artery will expose the artery and may lead to erosion of the vessel wall and the dire complication of a carotid blow out. Soft tissue coverage is necessary to prevent this dire complication, and Chan et al. [16] have described their experience in covering the exposed carotid artery with a free muscle flap, whereas Khoo et al. [17] published a report of two cases using a free radial forearm flap to cover the nasopharynx. In our experiment we demonstrated, by use of a cadaver, that it is possible, with this lateral palatal flap approach, to suture tissue to the nasopharynx with robotic instruments. The EndoWrist^®^ of the surgical robot made movements such as suturing possible; this is not possible with current endoscopic instruments.

The expected complication of the lateral palatal approach was healing of the incision and the risk of developing a dehiscence of the palatal wound and formation of palatal fistula. It is uncertain whether soft palate movement would be compromised by the division and resuturing of the palatoglossus muscle. In the cadaver, resection of the nasopharynx took approximately 30 min. For live patients, resection would be expected to take much longer, in order to control venous bleeding in the pterygoid plexus.

## Conclusion

In conclusion, we have described the palate flap approach for robot-assisted resection of the nasopharynx. The approach enabled en bloc resection of nasopharyngeal mucosa, the cartilaginous Eustachian tube, the adjacent parapharyngeal fat, and the medial pterygoid muscle. The approach enabled excellent visualization of the lateral wall of the nasopharynx and the parapharyngeal soft tissue. Use of the robot also enabled suturing of a fasciocutaneous flap into the nasopharynx for coverage of the defect after resection.
